# Addressing Barriers to Autopsy and Genetic Testing in Stillbirth Workup

**DOI:** 10.1097/og9.0000000000000025

**Published:** 2024-08-01

**Authors:** Karen J. Gibbins, Neeta L. Vora, Akila Subramaniam, Jessica M. Page, Naomi O. Riches, Erin Rothwell

**Affiliations:** Division of Maternal-Fetal Medicine, Department of Obstetrics and Gynecology, Oregon Health & Science University, Portland, Oregon; the Division of Maternal-Fetal Medicine, Department of Obstetrics and Gynecology, University of North Carolina at Chapel Hill, Chapel Hill, North Carolina; the Division of Maternal-Fetal Medicine, Department of Obstetrics and Gynecology, University of Alabama at Birmingham, Birmingham, Alabama; and the Department of Obstetrics and Gynecology, Intermountain Health, and the Department of Obstetrics and Gynecology, University of Utah, Salt Lake City, Utah.

## Abstract

The cause of death in many stillbirths remains unexplained, in part because of systematic barriers to complete workup. We review the importance of placental pathology, fetal autopsy, and genetic testing in stillbirth workup. Placental pathology is useful in 65% of stillbirths, and fetal autopsy is useful in 42%. Currently, only 21% of stillbirths in the United States undergo autopsy. Barriers to complete stillbirth workup include cost of autopsy and genetic testing, availability of perinatal pathology expertise, health care professional knowledge about workup, and availability of skilled counseling to support bereaved parents in making decisions about autopsy and genetic workup. We propose solutions to overcome these barriers, including policy changes to expand access and a framework for high-quality patient counseling and decision support.

## Abstract 2

Practice and policy interventions are needed to improve access to and uptake of autopsy and genetic testing in stillbirth workup.

Stillbirth is one of the most common severe pregnancy complications. In 2021, the United States stillbirth rate was 5.73 fetal deaths per 1,000 births after 20 weeks of gestation.^1^ Causes of stillbirth include medical complications such as diabetes, obstetric disorders such as preterm birth, hypertensive diseases, placental dysfunction, umbilical cord complications, genetic causes, and infections, and some remain unexplained.^2^

Unexplained stillbirth remains common, meaning the cause of death cannot be determined.^2^ In some cases, a complete workup has been performed, and no cause can be identified with currently available testing. Other times, the reason is that the search for the cause was incomplete. Determining cause of death is important for grieving parents and to guide future reproductive plans because some causes have high risks of recurrence. Given the wide range of causes, understanding the causes of stillbirth is paramount to any population-based initiatives to reduce preventable stillbirth. A brief overview of the tests recommended to determine cause of death in stillbirth is presented in Figure 1.^3,4^

**Fig. 1.**
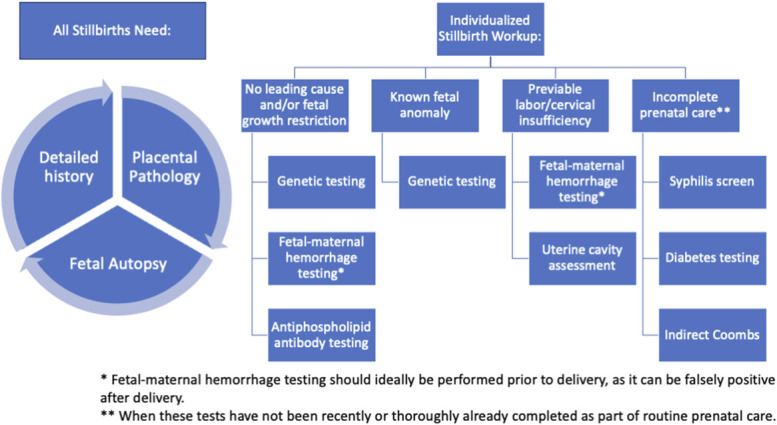
Elements of recommended stillbirth workup.

*Gibbins. Barriers to Stillbirth Workup. O&G Open 2024*.

Placental pathology is the single most useful test in stillbirth workup, closely followed by fetal autopsy and genetic testing.^4^ Unfortunately, there are multiple barriers to pathology and genetic testing, leading to incomplete workups in most stillbirths in the United States.^5^ In this narrative review, we discuss the importance of pathology and genetic testing. We review barriers to completion of this testing and suggest actions to overcome these barriers, and we highlight best practices in counseling bereaved parents about their testing options.

## IMPORTANCE OF PATHOLOGY

Most experts, including the American College of Obstetricians and Gynecologists, emphasize the importance of placental pathology and fetal autopsy in stillbirth workup.^3,6^ These two tests have the highest utility in determining cause of death. Placental pathology provided useful information in 65% of cases and fetal autopsy in 42% in the SCRN (Stillbirth Collaborative Research Network) study, a population-level assessment of 512 stillbirths.^4^ Unfortunately, despite being one of the most useful tests in the stillbirth workup, autopsy completion for stillbirth has decreased over time in the United States, from 35%–48% in the late 1990s to only 21% in 2014–2016.^5^

There is variation in autopsy uptake by setting. In a study of all stillbirths in Arkansas from 2015 to 2020, only 13% of cases underwent autopsy,^7^ even lower than national averages. In the SCRN study, in which trained research staff offered autopsy and genetic testing to all families at no cost, 84.5% of parents chose full autopsy.^8^ Globally, information on consent rates is limited. France had overall rates of consent for fetal autopsy of 70.8–75.3% from 2005 to 2014,^9^ with lower rates in a disadvantaged district (39.1% in 2014) even with the cost fully covered.^10^ In the United Kingdom and Australia, parents are almost always offered an autopsy (98% and 95%, respectively), yet only 53% and 43% are completed.^11,12^ The situational determinants that either support or act as barriers to autopsy are not well established.

## IMPORTANCE OF GENETIC TESTING

Genetic testing is a key part of stillbirth workup.^4^ An abnormal karyotype is found in 6–13% of stillbirths and more than 20% of stillbirths with structural anomalies. Chromosomal microarray (CMA) has increased yield over traditional karyotype in stillbirth,^13,14^ because CMA can be performed with direct DNA, whereas karyotype requires cultured cells. In the SCRN study, not only was CMA more likely than karyotype to yield an interpretable result (87.4% vs 70.5%, *P*<.001), but CMA was more likely to detect genetic abnormalities, either aneuploidy or pathogenic copy number variants (8.3% vs 5.8%, *P*=.007).^14^

Whole-genome sequencing and whole-exome sequencing also play a role in stillbirth workup.^15^ When 246 stillbirths from SCRN with negative karyotype or CMA underwent whole-exome sequencing for pathogenic variants linked to stillbirth, the diagnostic yield of whole-exome sequencing was 6.1%, or 15 of 246 cases. Ab additional six cases had a suggestive genotype for stillbirth (totaling 21 of 246 cases, or 8.5%) beyond karyotype or CMA.^16^ It is important to note that all cases in this analysis had complete autopsy information and thus informative phenotypic information. Cases with one or more structural anomalies had increased odds of a molecular diagnosis (odds ratio 8.8, 95% CI, 1.7–38.4). Considering the increased recurrence risk with genetic abnormalities that are not de novo, these results are important for both identifying cause of death in index stillbirths and providing preconception counseling and care in any subsequent pregnancy. Identifying a genetic cause allows prenatal diagnosis or preimplantation genetic diagnosis in future pregnancies. Although whole-exome sequencing and whole-genome sequencing are not currently recommended as first-line tests, they are reasonable to consider in stillbirths that remain unexplained after CMA.

## BARRIERS TO COMPLETION OF THE WORKUP AND PROPOSED SOLUTIONS

Modifiable barriers to complete stillbirth workup include availability of pathologists trained in stillbirth evaluation, cost of autopsy and genetic testing, and optimized counseling that supports shared decision making on fetal autopsy. Some of these require systems-level change; others can be improved with individual practice.

### Barrier: Limited Availability of Perinatal Autopsy

Both placental pathology and fetal autopsy require unique pathology expertise. Although it is not required that studies be performed by fellowship-trained perinatal pathologists, it is necessary that the pathologist have expertise in stillbirth evaluation. Perinatal pathology can be part of pediatric pathology training or its own separate fellowship. As of 2019, there were 268 pediatric pathologists in the United States,^17^ with only a subset with expertise in perinatal pathology and employed in a hospital offering obstetric care.

Patients with stillbirths commonly present at their local or regional hospitals, which are not necessarily academic or tertiary care centers with perinatal pathology experts. Thus, many patients deliver their stillborn fetuses at facilities without local perinatal pathology expertise. In this situation, the family has three options: 1) not pursuing autopsy, 2) pursuing autopsy with a pathologist who may lack the necessary expertise to yield best results, and 3) coordinating transfer of the fetus to a center that has the necessary expertise.

### Action: Increase Regional Access to Perinatal Autopsy

We encourage individual hospitals to determine whether the necessary expertise for postmortem examinations is available locally. If not available, we encourage collaborating with facilities that do have pathologists with such expertise to develop a protocol for transfer of the stillborn fetus to the facility where autopsy can be completed. This protocol should include a checklist of necessary documentation, logistics of transportation, anticipated timeline until the fetus is returned to the funeral home, and key contacts at both local and referral hospitals. Laws regarding transport of a stillborn fetus vary by state and should be incorporated. Ideally, this would be organized at a regional level to minimize burden to individual hospitals. Admittedly, the time required for transfer causes a small delay in time to autopsy, which may increase the degree of tissue necrosis. If parents are resistant to transport of the fetus, there is still high utility of transporting the placenta to a center with a skilled perinatal pathologist. It is also possible for a perinatal pathologist to review slides originally obtained at the delivery hospital to offer expert opinion in situations in which this may help to inform cause of death.

### Action: Optimize Availability of DNA From Stillbirth

All patients should be counseled about the utility of genetic testing in determining possible causes of stillbirth.^14,16,18^ Amniocentesis is the preferred method of obtaining fetal DNA because of the lower success of culture of cells from postdelivery tissue.^19^ However, if a patient declines amniocentesis or if there are other barriers to amniocentesis, other tissues from delivery can be used instead^20–25^ (Table 1). All of these specimen types can readily be sent to commercial laboratories for genetic testing. If an autopsy is performed, fetal DNA can also be obtained by the pathologist from fetal tissues.

**Table 1.** Methods of Obtaining Samples for Genetic Testing

Sample	How to Obtain	Notes
Amniocentesis	• Standard amniocentesis procedure before delivery	• Preferred
Umbilical cord blood	• Aspirated from umbilical cord at delivery	• Only possible with very recent death, eg, intrapartum stillbirth
	• EDTA lavender top tube and green top sodium heparin tube	
Placental tissue	• 1-cm^2^ specimen from fetal side, excised with a scalpel	• Requires maternal blood sample for ruling out maternal cell contamination, 3–5 mL in EDTA lavender top tube
	• Include chorionic plate	• Second choice after amniocentesis sample
	• Can be taken below cord insertion site	
	• Can also be products of conception from dilation and evacuation procedure	
	• Place sample in sterile saline or sterile medium from laboratory; no fixative	
	• Keep at room temperature	
Umbilical cord	• 1.5-cm segment	• Infrequently used
Fetal tissue	• Internal tissue sample	• When maceration is present, placental tissue is most useful
	• Achilles tendon, costochondral junction, or patella	• Some laboratories prefer skin because skin grows better than tendon, but this is becoming less common
Paraffin blocks of fetal or placental tissue	• From stored samples	• Use only when fresh samples were not obtained
		• Available only at limited cytogenetics laboratories

If fetal tissue (eg, amniotic fluid, placenta) is obtained, we recommend the following diagnostic testing algorithm. Chromosomal microarray yields results more often than karyotype analysis.^14^ However, most stillbirths attributable to a genetic abnormality are still the result of the most common aneuploidies (trisomy 18, 13, 21; monosomy X). We therefore recommend karyotype (20 cells) with reflex to CMA. In the case of already-performed low-risk cell-free DNA for aneuploidy testing, it is reasonable to start with CMA. Pretest counseling for CMA should include the following: CMA 1) is more costly; 2) can result in variants of uncertain significance^26^ (1–2% of the time); 3) can reveal low-penetrance, adult-onset conditions and consanguinity; and 4) cannot detect mechanism of aneuploidy (nondisjunction vs robertsonian translocation).

Whole-exome sequencing and whole-genome sequencing are also available from clinical laboratories for determination of the cause of stillbirth when microarray is nondiagnostic.^27^ Given the complex pretest and posttest counseling needed for sequencing, a genetic counselor should be included when sequencing is planned. It is important to note that a fetal autopsy that provides robust phenotypic information could improve interpretation of single nucleotide variants on whole-exome sequencing and whole-genome sequencing.^27^ After genetic testing, a genetic counseling appointment should ideally be made to discuss the results of genetic testing with the parents. Because access to genetic counselors can be limited, referral to tertiary centers for this counseling may be necessary.

### Barrier: Cost

Stillbirth and neonatal death are expensive. One analysis found that stillbirth delivery costs alone were $757 (95% CI, 201–1,312, *P*<.001) more than a live birth.^28^ There are also the costs of additional tests and of funeral and burial expenses. Despite being a first-line test in the workup of stillbirth, fetal autopsy is not covered by insurance. Medicare and Medicaid laws do not currently allow coverage of autopsies, and many private insurances do not cover this cost. Costs of autopsy range from $1,500 to $5,000 when paid out of pocket.^29,30^ This is prohibitive for most people. When not covered or partially covered by insurance, out-of-pocket costs for genetic testing can range from $100 to more than $2,000,^31^ depending on the test. Thus, comprehensive workup of stillbirth is cost prohibitive for many parents. Both access to an expert pathologist and the ability to pay for testing represent sources of inequity in care for families who experience stillbirth.

### Solution: Cost

Some hospital systems choose to absorb the cost of fetal autopsy and offer it free of charge. However, this is far from universal, and pregnant people typically will not know this about their chosen hospital until after delivery. Although stillbirth is far from rare, with state rates ranging from 11.1 stillbirths per 1,000 live births in Mississippi to 3.4 per 1,000 in New Mexico,^32^ the hospital cost of covering autopsy remains small. A hospital with 2,000 births per year may have 7–22 stillbirths. If all bereaved parents chose autopsy and assuming a cost of $2,000 per autopsy, these would equal a hospital-wide cost of only $14,000–$44,000 annually.

On a systems level, we advocate for change in insurance policy to cover perinatal autopsy given its importance in stillbirth evaluation. Such advocacy should also recommend coverage of, at minimum, CMA for genetic testing and ideally whole-exome sequencing and whole-genome sequencing when clinically indicated.

### Barrier: Counseling

Skilled counseling is necessary to support parents in deciding whether to pursue autopsy for their stillborn fetus. Most bereaved parents have never considered this decision, one that is time sensitive in a moment of acute trauma. Parents deserve thorough, compassionate counseling in a manner that supports shared decision making. Unfortunately, this does not always happen. One Italian survey of 187 parents with a stillbirth found that only 64.5% discussed autopsy at all with a clinician and 28.3% received a shared decision making approach.^33^ It is important to note that, when parents decline autopsy, some regret the decision in the future.^34,35^

To provide meaningful counseling for bereaved parents, it is important to understand what factors motivate parents to choose autopsy. A study of 19 bereaved parents (11 of whom chose fetal autopsy) found that motivations to pursue autopsy included a need for information, a sense of altruism, and a belief in science.^34^ Some parents particularly appreciated having written information to review. As of now, according to a Cochrane review, there are no published interventions to support this process.^36^ Another qualitative study found that when parents were undecided about autopsy, decisional drivers included the clinician's initial approach to the conversation, time for deliberation, detailed discussion about the procedure, and formal consent.^33,37–39^ These patient perspectives highlight the importance of high-quality resources and counseling to optimize decision making on autopsy.^40^

### Solutions: Counseling

We suggest the following steps to ensure that every family receives counseling and decision support, the right person performs the counseling, and the counseling each family receives is thorough and of high quality.

1. Every family receives counseling and decision support.

Every family must be offered counseling about the utility of autopsy. Not all information can or should be presented at the time of initial diagnosis of stillbirth, potentially leading to incomplete counseling. We suggest adding counseling to any existing institutional checklist for stillbirth and perinatal death care. Proposed checklist items include both “counseling regarding autopsy” and “parents' decision regarding autopsy confirmed: yes/no/other” because these are separate items requiring multiple discussions.

Bereavement navigators can also help ensure that counseling happens and is readdressed as needed. A bereavement navigator serves as the family's point of contact. They may assist with the logistics of obtaining a fetal death certificate, coordinating burial or cremation, and arranging follow-up. They may also provide support in memory making and memento creation such taking photos and making molds of hands and feet. Although this is not a licensed role, it is often filled by a nurse, and the Association of Women's Health, Obstetric, and Neonatal Nurses offers a Perinatal Bereavement Certificate Program along with resources to help build a bereavement program.^41^

2. The right person performs the counseling.

Stillbirth remains both devastating and rare enough to be jarring for most clinicians. Many people fear upsetting bereaved parents by discussing anything with the families, let alone autopsy.^35^ This hesitancy is perceived by many parents as cold and absent communication.^42^ Moreover, many clinicians are not confident in their knowledge of autopsy, and educational materials to support this discussion are lacking.^43^

The right person to perform counseling about stillbirth workup and autopsy is someone who is skilled and comfortable with patient-centered conversations about death, knowledgeable about the utility and limitations of autopsy, and able to describe the steps of autopsy and different options for autopsy available. If one individual has this expertise and another has an emotional connection with the patient, both can counsel together, which also serves to educate the less experienced clinician. Tools to support parents' decisions may also be beneficial during counseling. The government of South Australia^44^ has developed a patient-facing information sheet that is specific to the medical system of South Australia. There are currently no formal decision aids to support parents' decisions about autopsy,^36^ although one is under development.^34^

3. Counseling is thorough and of high quality.

Table 2 outlines high-quality counseling on autopsy and genetic testing for stillbirth workup and was developed with the SPIKES (setting up, perception, invitation, knowledge, emotions with empathy, and strategy or summary) method of delivering bad news from the oncology field.^45^ Continued medical education may be necessary to build a workforce capable of providing optimal counseling.

**Table 2.** Counseling Families About Stillbirth Workup Using the SPIKES Model

SPIKES Framework	Key Points of Optimal Counseling
S: Setting up the discussion	• Prepare for the discussion. Run through your plan before engaging the patient.
	• Establish the setting. This counseling should be done in a private space when the pregnant person is not in physical distress or sedated. Ensure that all key participants are present according to patient preferences. This may include a partner or other key family members or support people.
	• Situate yourself in the space. Sit down and greet everyone present.
	• Ensure that you are unlikely to be called away mid-discussion. This may be less feasible if you are the on-call clinician in the labor and delivery department, but communication with other clinicians on the floor may support this goal.
P: Assessing the patient's perception	• In oncology, this means asking the patient what their understanding of the medical situation is. For a family with a recently diagnosed stillbirth, this framework is inappropriate because you are essentially asking the family to say, “Our baby is dead.” Instead, ask if anyone has talked to them or if they know anything about tests that can be done to discover why their baby died.
I: Obtaining patient's invitation	• State that a number of tests are recommended to help find this answer, including examinations of the placenta and the baby's body, also called an autopsy, as well as genetic testing. Ask if now is an okay time to talk about these options or other parts of the possible workup.
	• If parents say that now is not an okay time, inquire if you can check back later.
K: Giving knowledge and information to the patient	• Frame autopsy as a part of ongoing care for their baby, explaining that it is useful in determining cause of death in 40% of cases. Explain that examination of the placenta is useful in more than 60% of cases. Together, autopsy and placental examination are the most useful tests to determine cause of death.
	• State that national guidelines (ACOG) recommend autopsy for workup of all stillbirths. Explain that the information found with autopsy can be used to both learn cause of death and guide care in future pregnancies. Explain that sometimes stillbirth happens for random reasons but that sometimes it is attributable to causes that have a risk of happening again in future pregnancies.
	• Acknowledge that completing an autopsy does not ensure that cause of death will be discovered.
	• Explain steps of the autopsy, emphasizing that the highly trained pathologists who provide this care do so with the utmost respect. Explain that, because there are no incisions placed on the face, an open-casket funeral remains a possibility if that is desired. A hat can cover any incisions made to the head, or head incisions can be declined.
	• Explain that CMA is considered first line for genetic testing in the setting of otherwise unexplained stillbirth. Tell parents that CMA finds an abnormality in 8% of stillbirths. Offer to help determine insurance coverage and cost of CMA for your patient. If your patient is undecided about CMA, give options for storing DNA for future analysis.
	• If available, provide written material for parents to review during their deliberation. A decision aid would be particularly useful here.
E: Addressing patient's emotions with empathic responses	• A wide range of emotional responses are normal during this conversation. Some parents display their grief openly, and some approach this conversation with a degree of intellectualization and can appear somewhat flat in affect. Remember that they have just experienced an acute trauma and that there are many ways to process. You can respond to emotions communicated by acknowledging and validating them.
	• Throughout the discussion, it is appropriate to express empathy, such as saying, “I'm so sorry we are having this difficult discussion,” or “I know it is extremely difficult to think about this happening for your baby. Is it ok to keep talking, or should we take a break?”
	• If there are religious concerns about autopsy, offer to consult with religious leaders.
S: Strategy and summary	• Give space for deliberation. Determine a plan for returning to discuss the family's decision or to answer any additional questions.
	• If families are ready to decide and choose to pursue autopsy, provide formal consent along with a timeline for when results are to be expected and how they will be delivered. In the era of immediate-release results, explain that autopsy and genetic testing results will be available on their patient portal as soon as they are available to the clinician. Thus, it is likely the patient will see them first. Ask how they want to be contacted to discuss these and if they would like to schedule an appointment to do so in advance.

ACOG, American College of Obstetricians and Gynecologists; CMA, chromosomal microarray.

## ALTERNATIVES TO CONVENTIONAL AUTOPSY

Conventional autopsy is not the right choice for every family.^46^ Parents who decline autopsy often express a goal of protecting their baby from additional harm.^34^ Acceptability of autopsy also varies by cultural and religious background.^47^ People who decline conventional autopsy after adequate counseling should be offered alternatives. A retrospective survey of 859 bereaved parents suggested that, although 54% would consent to standard autopsy, 91% would consent to less invasive forms of autopsy.^48^

X-ray may be useful if the primary abnormality is skeletal.^49^ Microfocal computed tomography and magnetic resonance imaging are more useful and have reasonable concordance with traditional autopsy^50,51^ in the range of 80–95%, with strongest performance for renal, neural, and cardiac abnormalities^52,53^; computed tomography and magnetic resonance imaging are less useful for abdominal and lung findings.^54^ Magnetic resonance imaging may be more useful than traditional autopsy for intracranial abnormalities given the instability of brain tissue after death. However, these modalities require workflows, access to specialized equipment, and experienced radiologists.

Partial autopsy is also an option,^55^ which allows parents to decline head incisions but permit abdomen and chest autopsy. They also could elect for percutaneous or laparoscopic biopsies to permit histopathologic assessment of key organs and tissues.^56,57^ Although this has limited utility compared with traditional autopsy, it is more informative than external inspection alone.

For all cases, documentation should be detailed, including chronology of events and labor course. An external examination of the placenta and fetus in the delivery room is useful for all cases, but particularly if parents have declined fetal autopsy. The placenta should be weighed, and the length of the umbilical cord should be measured. Photographs of the placenta, umbilical cord, and fetus should be documented in the medical record with the parents' permission. Any fetal anatomic abnormalities should be noted. Fetal measurements should include weight, length, and head circumference.^3^ These details help future review and attempts to establish cause of death, even if parents do not pursue autopsy.

## SUMMARY

Thorough stillbirth evaluation is important to understanding the causes of stillbirth and reducing incidence, yet there are many barriers. In some situations, autopsy may not be the right choice for families. If cause of death is already confidently determined, autopsy will not provide additional yield (eg, when prenatal imaging and genetic testing are conclusive or if stillbirth is attributable to intrapartum previable death in the setting of cervical insufficiency). For some families, their values and beliefs about mortality and care of a dead body preclude autopsy as an option, regardless of the utility.^47^ Our goal is to ensure that all bereaved parents have the information and support to make the best decision for them. For some of these families, a combination of placental pathology, external examination, and genetic testing may be sufficiently diagnostic. As obstetric clinicians, we owe our patients adequate access and counseling about their options for a full stillbirth workup. Within this text, we have outlined a pragmatic approach to improve this care. Some of these recommendations can be undertaken on the individual level; others require systems-level change, which will require collective effort to achieve.
